# Association Between Sleep Duration and Ideal Cardiovascular Health Among US Adults, National Health and Nutrition Examination Survey, 2013–2016

**DOI:** 10.5888/pcd17.190424

**Published:** 2020-06-11

**Authors:** Rebecca E. Cash, Chloe M. Beverly Hery, Ashish R. Panchal, Julie K. Bower

**Affiliations:** 1 The Ohio State University College of Public Health, Division of Epidemiology, Columbus, Ohio; 2 National Registry of Emergency Medical Technicians, Columbus, Ohio; 3 Massachusetts General Hospital, Department of Emergency Medicine, Boston, Massachusetts; 4 The Ohio State University Wexner Medical Center, Department of Emergency Medicine, Columbus, Ohio

## Abstract

**Introduction:**

Short or long sleep duration is a risk factor for cardiovascular disease, but the association between sleep duration and cardiovascular health is unclear. Our objective was to quantify the association between sleep duration and ideal cardiovascular health (CVH) in US adults. We hypothesized that very short (<6 h) and very long (≥9 h) sleep duration were associated with poorer CVH compared with sleep lasting 7 to <8 hours.

**Methods:**

We conducted a cross-sectional evaluation of the nationally representative National Health and Nutrition Examination Survey in 2 cycles (2013–2014 and 2015–2016). Participants were 7,784 cardiovascular disease–free US adults aged 20 to 75. Self-reported sleep duration was categorized as <6 hours, 6 to <7 hours, 7 to <8 hours, 8 to <9 hours, and ≥9 hours. The American Heart Association’s ideal CVH metrics were used to determine the number of ideal CVH components, dichotomized as ideal (5–7 components) or not ideal (0–4 components). Survey-weighted logistic and linear regression models were used to determine the association between sleep duration and ideal CVH.

**Results:**

The weighted prevalences of those who slept 7 to <8 hours were 30.4%, very short sleep duration (<6 h), 9.0%, and very long duration (≥9 h), 13.5%. Only 21.3% of the population had ideal CVH. Compared with 7 to <8 hours, very short duration (OR = 0.65; 95% confidence interval [CI], 0.47–0.90) and very long duration (OR = 0.72; 95% CI, 0.55–0.94) were associated with decreased odds of ideal CVH. We confirmed findings by using linear regression.

**Conclusions:**

Very short and very long sleep duration were associated with decreased odds of ideal CVH and lower mean CVH scores. Future research should focus on clarifying causal associations between sleep duration and ideal CVH.

**Summary**

**What is already known on this topic?**

Short and long sleep durations are risk factors for cardiovascular disease, but limited work has assessed the association between sleep duration and cardiovascular health, a measure of the health of the cardiovascular system. Ideal cardiovascular health is associated with a reduced risk of developing cardiovascular disease.

**What is added by this report?**

By using a nationally representative sample of US adults from the National Health and Nutrition Examination Survey, we showed that very short (<6 h) and very long (≥9 h) sleep duration was significantly associated with decreased odds of ideal cardiovascular health and a significant decrease in mean cardiovascular health score.

**What are the implications for public health practice?**

Sleep may be an additional component to describe health of the cardiovascular system. Unhealthy sleep duration represents a modifiable risk factor that can be targeted for population-level intervention.

**MEDSCAPE CME**

Medscape, LLC is pleased to provide online continuing medical education (CME) for this journal article, allowing clinicians the opportunity to earn CME credit.

In support of improving patient care, this activity has been planned and implemented by Medscape, LLC and *Preventing Chronic Disease*. Medscape, LLC is jointly accredited by the Accreditation Council for Continuing Medical Education (ACCME), the Accreditation Council for Pharmacy Education (ACPE), and the American Nurses Credentialing Center (ANCC), to provide continuing education for the healthcare team.

Medscape, LLC designates this Journal-based CME activity for a maximum of 1.00 *AMA PRA Category 1 Credit(s)™*. Physicians should claim only the credit commensurate with the extent of their participation in the activity.

All other clinicians completing this activity will be issued a certificate of participation. To participate in this journal CME activity: (1) review the learning objectives and author disclosures; (2) study the education content; (3) take the post-test with a 75% minimum passing score and complete the evaluation at http://www.medscape.org/journal/pcd; (4) view/print certificate.

**Release date: June 11, 2020; Expiration date: June 11, 2021**

**Learning Objectives**

Upon completion of this activity, participants will be able to:

1. Assess the relationship between sleep duration and the risk for cardiovascular events
2. Distinguish the proportion of US adults getting the recommended hours of sleep
3. Analyze variables in the cardiovascular risk profile affected by sleep duration
4. Evaluate how sleep duration can affect the cardiovascular risk profile among adults

**EDITOR**

Rosemarie Perrin

**CME AUTHOR**

Charles P. Vega, MD

Health Sciences Clinical Professor of Family Medicine

University of California, Irvine, School of Medicine

Irvine, California 

Disclosure: Charles P. Vega, MD, has disclosed the following relevant financial relationships:

Served as an advisor or consultant for: Johnson & Johnson Pharmaceutical Research & Development, LLC; GlaxoSmithKline

Served as a speaker or a member of a speakers bureau for: Genentech, GlaxoSmithKline

**AUTHORS**

Rebecca E. Cash, PhD, MPH

Division of Epidemiology

The Ohio State University College of Public Health

Columbus, Ohio

The National Registry of Emergency Medical Technicians

Columbus, Ohio

Department of Emergency Medicine

Massachusetts General Hospital

Boston, Massachusetts

Disclosure: Rebecca E. Cash, PhD, MPH, has disclosed no relevant financial relationships.

Chloe M. Beverly Hery, MS

Division of Epidemiology

The Ohio State University College of Public Health

Columbus, Ohio

Disclosure: Chloe M. Beverly Hery, MS, has disclosed no relevant financial relationships.

Ashish R. Panchal, MD, PhD

Division of Epidemiology

The Ohio State University College of Public Health

Columbus, Ohio

The National Registry of Emergency Medical Technicians

Columbus, Ohio

Department of Emergency Medicine

The Ohio State University Wexner Medical Center

Columbus, Ohio

Disclosure: Ashish R. Panchal, MD, PhD, has disclosed no relevant financial relationships.

Julie K. Bower, PhD, MPH, FAHA

Division of Epidemiology

The Ohio State University College of Public Health

Columbus, Ohio

Disclosure: Julie K. Bower, PhD, MPH, FAHA, has disclosed the following relevant financial relationships:

Owns stock, stock options, or bonds from: Vertex Pharmaceuticals

Employed by a commercial interest: Vertex Pharmaceuticals

## Introduction

Approximately 60% of US adults report sleeping the recommended 7 to 9 hours per night (1,2). The consequences of unhealthy sleep, which include risk of chronic diseases, productivity loss, and fatigue-related performance concerns, have been described as a critical public health problem (3). Meta-analyses show a U-shaped association between short and long sleep duration and risk of developing cardiovascular disease (CVD) (4–6). Potential mechanisms for this association with short sleep include dysregulation of appetite hormones (7), glucose intolerance (4), the up-and-down regulation of gene expression (8), and circadian rhythm misalignment (9), which may result in inflammatory conditions and development of other CVD risk factors such as hypertension (10,11). The biologic mechanism of increased risk from long sleep is less clear and may be related to sleep fragmentation, depression, or underlying poor health (12).

Little evidence exists regarding the association between healthy sleep and a widely used metric for the general state of well-being of the cardiovascular system called ideal cardiovascular health (CVH). In 2010, the American Heart Association (AHA) defined ideal CVH on the basis of 7 modifiable factors: smoking, body mass index (BMI) (weight in kg divided by height in m^2^), diet, physical activity, blood pressure, fasting blood glucose, and total cholesterol (13). Each CVH component is scored as ideal, intermediate, or poor on the basis of established cut points (13). Meeting the ideal criteria for 5 to 7 components is associated with a reduced risk of developing CVD (14,15).

Sleep debt is associated with poor CVH (16), and very short sleep duration is associated with increased odds of CVD risk factors such as hypertension and obesity (17). Because the association between ideal CVH and sleep duration remains unclear, our objective was to quantify that association among a nationally representative sample of US adults. We hypothesized that very short (<6 h) and very long (≥9 h) sleep duration were associated with decreased odds of ideal CVH.

## Methods

### Data source, sample, and design

We conducted a cross-sectional analysis of data from the National Health and Nutrition Examination Survey (NHANES), a nationally representative cross-sectional survey of noninstitutionalized civilian residents of the United States, conducted continuously in 2-year cycles (18). NHANES uses a complex sampling strategy with a multistage stratified probability cluster design to achieve a nationally representative sample. The survey consists of in-home interviews and a medical examination of approximately 10,000 children and adults at each 2-year cycle. The medical examination component is voluntarily completed by a subset of all participants. We used 2 cycles of NHANES for our evaluation, data collected in 2013–2014 and 2015–2016. These de-identified data are publicly available.

A total of 10,068 adults aged 20 to 75 completed the medical examination component during the 2 cycles. Of those, 7,784 (77%) adults without a self-reported history of CVD (congestive heart failure, coronary heart disease, angina pectoris, myocardial infarction, or stroke), who had complete measurement of all cardiovascular health components and covariates were included in the analysis. A total of 834 respondents were excluded because of a history of CVD, 863 because they were missing CVH components, and 1,448 because they were missing covariates. Respondents could be excluded for more than 1 reason. The most common missing covariates were depression status (n = 590) and household income (n = 521).

### Measures

**Exposure: sleep duration**. Usual weekday or workday sleep duration was self-reported by participants. In 2013–2014, this information was elicited directly by asking for the usual hours of sleep on weekdays or workdays. In the 2015–2016 cycle, usual hours of sleep were calculated by asking respondents their normal bed time and wake time on weekdays or workdays. Sleep duration was categorized as <6 hours, 6 to <7 hours, 7 to <8 hours, 8 to <9 hours, and ≥9 hours.

**Outcome: cardiovascular health score.** Cardiovascular health was defined according to the AHA’s ideal CVH metrics (13). These metrics consist of 7 modifiable health behaviors and factors that were scored as ideal (2 points), intermediate (1 point), or poor (0 points) for adults, as described previously. Participants self-reported their smoking status, frequency of physical activity, and use of medications to control blood pressure, cholesterol, or diabetes mellitus. Dietary habits were reported through one or two 24-hour recalls (single day or average intake across both recalls). Ideal diet was assessed with the AHA Healthy Diet Score (13) by measuring the intake of fruits and vegetables, whole grains, sodium, fish, and sugar-sweetened beverages. Total cholesterol, hemoglobin A_1c_, and blood pressure were measured by trained professionals as part of the NHANES medical examination component. Hemoglobin A_1c_ was used as a proxy for fasting plasma glucose (19,20); otherwise, we used the established CVH criteria (13). Briefly, the ideal criteria were never smoking or quit smoking more than 12 months ago; BMI of <25.0 kg/m^2^; meeting 4 to 5 of the AHA Healthy Diet Score components; physical activity of ≥150 minutes per week of moderate exercise, ≥75 minutes per week of vigorous exercise, or ≥150 minutes per week of moderate and vigorous exercise; blood pressure of <120 mm Hg/<80 mm Hg without medication; total cholesterol of <200 mg/dl without medication; and hemoglobin A_1c_ of <5.7% without medication. The total score and number of ideal categories met by each participant were summed, and the resulting CVH score was analyzed as both continuous and categorical. For our study, overall ideal CVH was defined as meeting ideal criteria for 5 to 7 components, intermediate CVH as 3 to 4 components, and poor overall CVH as meeting 0 to 2 components. For logistic regression models, we further dichotomized the overall CVH score to ideal (5–7 categories) or not ideal (0–4 categories).

**Covariates.** Covariates were selected a priori based on prior literature and substantive reasoning. Participant age, sex, race/ethnicity, education level, and marital status were self-reported. The ratio of monthly family income to poverty was used as a measure of socioeconomic status, categorized as ≤1.30, >1.30 to ≤1.85, and >1.85. This measure equates to the percentage above or below the federal poverty guidelines. Depression status was measured by the 9-item Patient Health Questionnaire depression screening instrument (21). The questionnaire scores items on a behavioral frequency scale and then dichotomizes them to none or moderate depression (0–14 points) and moderately severe to severe depression (15–27 points). Binge alcohol use was defined as drinking ≥4 alcoholic drinks per day for women or ≥5 for men, 12 or more times a year. Use of prescription sleep aids was self-reported. Reported current use of any of the following medications was considered use of sleep aid, regardless of frequency: amitriptyline, butabarbital, chloral hydrate, doxepin, estazolam, eszopiclone, flurazepam, mirtazapine, quazepam, ramelteon, temazepam, trazodone, triazolam, zaleplon, and zolpidem (22). Nonprescription sleep aid use was not documented.

### Statistical analyses

For population level estimates, a 4-year sample weight was calculated by dividing in half the mobile examination center’s sample analytic weights for the 2 cycles. Descriptive statistics were calculated, accounting for the complex survey design. We calculated unweighted frequency and weighted estimated population proportions or means. We also calculated the weighted population prevalence of each component and mean where appropriate and made comparisons by using Pearson’s χ^2^ statistic or the Wald test that adjusted for the survey design. Available cases were used as a sensitivity analysis and compared with estimates from the complete cases. Survey-weighted multivariable logistic regression and linear regression were used to estimate the association between sleep duration and ideal CVH, controlling for covariates. Separate models were constructed controlling for demographic characteristics (age, sex, race, race/ethnicity, education level, family income-to-poverty ratio) and social/clinical factors (depression status, alcohol use, prescription sleep aid use). A final fully adjusted model included all covariates. All analyses were conducted in STATA IC 15.1 (StataCorp LLC) at the α = 0.05 level.

## Results

The weighted mean age of the 7,784 participants included in our analysis was 44.5 (95% confidence interval [CI], 43.8–45.2) (Table 1). Approximately half of participants were women (51.5%; 95% CI, 50.3%–52.7%), and two-thirds were of non-Hispanic white race/ethnicity (65.5%; 95% CI, 59.9%–70.6%). About two-thirds (64.0%; 95% CI, 60.2%–67.6%) had monthly family incomes that were greater than 185% of the federal poverty guidelines. The weighted prevalence of moderately severe or severe depression was 2.6% (95% CI, 2.2%–3.1%), whereas 17.8% (95% CI, 16.1%–19.5%) reported binge drinking once a month or more. The weighted prevalence of prescription sleep aid use was 4.3% (95% CI, 3.5%–5.2%).

**Table 1:** Estimated Weighted Population Characteristics and Prevalence of Sleep and Cardiovascular Health Among US Adults Aged 20–75 (N = 7,784), National Health and Nutrition Examination Survey, 2013–2016

Characteristic	Unweighted, No.	Weighted^a^
**Age, mean, y**	7,784	44.5 (43.8–45.2)
**Sex**		
Male	3,704	48.5 (47.3–49.7)
Female	4,080	51.5 (50.3–52.7)
**Race/ethnicity**		
Non-Hispanic white	2,882	65.5 (59.9–70.6)
Non-Hispanic black	1,606	10.9 (8.5–13.9)
Hispanic	2,111	15.2 (11.7–19.4)
Non-Hispanic other	1,185	8.4 (7.1–10.1)
**Education level**		
High school diploma or less	3,247	33.4 (30.1–36.9)
Some college	2,447	33.3 (31.2–35.5)
College graduate or above	2,090	33.3 (29.4–37.5)
**Marital status**		
Married or coupled	4,760	64.9 (62.7–67.0)
Widowed, divorced, or separated	1,387	15.3 (13.9–16.7)
Never married	1,637	19.8 (18.0–21.8)
**Family income-to-poverty ratio**		
≤1.3	2,755	24.4 (21.4–27.6)
>1.3 to ≤1.85	1,082	11.7 (10.5–13.0)
>1.85	3,947	64.0 (60.2–67.6)
**Moderately severe or severe depression**	235	2.6 (2.2–3.1)
**Binge drinking once a month or more^b^**	1,217	17.8 (16.1–19.5)
**Prescription sleep aid use**	267	4.3 (3.5–5.2)
**Sleep duration, h**		
<6	862	9.0 (8.2–9.9)
6 to <7	1,571	18.5 (17.3–19.8)
7 to <8	2,135	30.4 (28.9–31.9)
8 to <9	2,112	28.6 (27.5–29.7)
≥9	1,104	13.5 (12.3–14.7)
**Overall CVH score**		
Mean	6,985^c^	8.0 (7.9–8.1)
Ideal (5–7 components)	1,156	17.8 (16.3–19.4)
Intermediate (3–4 components)	2,947	42.1 (40.9–43.3)
Poor (0–2 components)	3,200	40.1 (38.4–41.9)

Abbreviations: CI, confidence interval; CVH, cardiovascular health.

a Values are percentage (95% confidence interval) unless otherwise indicated. Because of survey weighting, proportions differ from calculations based on the unweighted number. Percentages may not total to 100% because of rounding.

b Binge drinking was defined as more than 4 drinks per day for women or more than 5 drinks per day for men.

c Mean score excluded those who were missing 1 or more CVH components.

The weighted prevalence of those who slept 7 to <8 hours was 30.4% (95% CI, 28.9%–31.9%), and the weighted prevalence of very short sleep (<6 h) was 9.0% (95% CI, 8.2%–9.9%) and very long (≥9 h) sleep was 13.5% (95% CI, 12.3%–14.7%). Ideal CVH was reported by only 17.8% (95% CI, 16.3%–19.4%) of the population, and nearly half were classified as intermediate overall CVH (Table 1). Differences were significant in the weighted prevalence of the overall CVH score and in several of the CVH components when stratified by the reported sleep duration (Table 2). The proportion of respondents who met the ideal criteria was highest with sleep durations of 7 to <9 hours for smoking, physical activity, and hemoglobin A_1c_. Results were not materially different using available case analysis.

**Table 2:** Weighted Mean and Population Prevalence of Overall CVH Score and Individual Components Stratified By Sleep Duration Among US Adults Aged 20–75 (N = 7,784), National Health and Nutrition Examination Survey, 2013–2016

Component	Definition^a^	Weighted Sleep Duration, mean or % (95% CI)					*P *Value^b^
		<6 h	6 to <7 h	7 to <8 h	8 to <9 h	≥9 h	
**Overall CVH score^c^**							
**Mean**	NA	7.4 (7.2–7.6)	7.9 (7.8–8.0)	8.2 (8.1–8.4)	8.1 (7.9–8.3)	7.7 (7.4–8.0)	<.001
Ideal	5–7	11.1 (8.3–14.7)	17.9 (15.9–20.0)	19.1 (17.1–21.3)	20.1 (17.8–22.6)	15.5 (12.7–18.7)	.01
Intermediate	3–4	41.2 (36.0–46.6)	41.5 (38.5–44.7)	43.6 (40.6–46.7)	41.9 (39.3–44.7)	41.0 (37.6–44.5)	
Poor	0–2	47.8 (43.0–52.6)	40.6 (37.9–43.3)	37.3 (34.4–40.2)	38.0 (35.1–41.0)	43.6 (39.4–47.8)	
**Smoking**							
Ideal	Never smoker or quit ≥12 months ago	47.9 (41.7–54.2)	56.0 (52.2–59.8)	63.2 (60.5–65.7)	60.3 (57.1–63.4)	57.0 (52.5–61.4)	<.001
Intermediate	Smoked ≥100 cigarettes and quit <12 months ago	21.6 (17.7–25.9)	21.6 (18.8–24.8)	22.0 (19.4–24.7)	23.7 (21.3–26.2)	19.2 (16.4–22.3)	
Poor	Current smoker	30.6 (25.6–36.1)	22.4(19.4–25.7)	14.9 (12.7–17.5)	16.0 (13.7–18.7)	23.8 (20.3–27.8)	
**Body mass index (kg weight/height in m^2^)**							
Mean	NA	30.6 (30.1–31.1)	29.6 (29.0–3.2)	29.1 (28.6–29.5)	29.0 (38.4–29.5)	29.0 (28.4–29.7)	<.001
Ideal	<25.0 kg/m^2^	23.3 (19.3–27.8)	27.0 (23.9–30.4)	30.1 (27.6–32.8)	30.4 (26.5–34.6)	31.2 (27.3–35.3)	.03
Intermediate	25.0–29.9 kg/m^2^	30.1 (26.0–34.7)	32.6 (29.6–35.7)	32.9 (30.1–35.9)	32.7 (30.2–35.3)	30.3 (27.0–33.9)	
Poor	≥30.0 kg/m^2^	46.6 (41.6–51.6)	40.4 (37.0–43.8)	37.0 (34.2–39.8)	36.9 (33.2–40.8)	38.5 (34.0–43.3)	
**Diet^d^**							
Ideal	4–5 components	0	0	0	0	0.1 (0.0–0.6)	.10
Intermediate	2–3 components	22.3 (17.2–28.3)	24.4 (21.2–28.0)	26.8 (23.9–29.9)	25.7 (22.7–28.9)	20.2 (17.4–23.2)	
Poor	0–1 components	77.7 (71.7–82.8)	75.6 (72.0–78.8)	73.2 (70.1–76.1)	74.3 (71.1–77.3)	79.7 (76.6–82.5)	
**Physical activity, min/wk **							
Mean	NA	168.9 (144.0–193.7)	165.9 (150.8–180.9)	165.6 (147.2–184.0)	171.8 (154.1–189.5)	166.6 (136.0–197.2)	.98
Ideal	≥150 min moderate and/or vigorous or ≥75 min vigorous	34.0 (29.7–38.6)	39.1 (35.8–42.5)	43.1 (39.3–47.0)	41.9 (38.4–45.5)	37.2 (32.0–42.7)	.03
Intermediate	1–149 min moderate and/or vigorous or 1–74 min vigorous	17.1 (14.3–20.3)	16.7 (13.9–20.0)	16.3 (14.2–18.7)	19.2 (17.0–21.7)	14.6 (11.7–18.1)	
Poor	None	49.0 (45.3–52.7)	44.2 (40.5–47.9)	40.6 (37.5–43.8)	38.9 (35.3–42.6)	48.2 (42.9–53.6)	
**Blood pressure, mm Hg**							
Systolic, mean	NA	122.3 (120.7–123.8)	121.3 (120.2–122.4)	120.5 (119.6–121.4)	121.3 (120.4–122.2)	121.8 (120.3–123.2)	.25
Diastolic, mean	NA	71.9 (70.8–73.1)	70.9 (70.1–71.7)	70.6 (69.8–71.4)	70.5 (69.6–71.3)	70.3 (69.1–71.5)	.16
Ideal	<120/<80 untreated	43.7 (39.5–48.0)	44.0 (40.7–47.3)	47.1 (43.6–50.6)	46.7 (44.2–49.2)	42.0 (38.3–45.8)	.24
Intermediate	SBP 120–139 or DBP 80–89 or treated to goal	46.0 (41.6–50.5)	48.3 (45.2–51.5)	45.5 (42.1–49.0)	45.0 (42.7–47.2)	48.1 (43.4–52.8)	
Poor	SBP ≥140 or DBP ≥90	10.3 (7.1–14.8)	7.7 (6.2–9.6)	7.4 (6.3–8.7)	8.3 (6.7–10.3)	9.9 (7.0-13.9)	
**Total cholesterol, mg/dl**							
Mean	NA	191.9 (187.6–196.3)	189.6 (186.6–192.7)	192.1 (190.0–194.2)	195.4 (192.1–198.7)	193.0 (188.5–197.4)	.07
Ideal	<200 untreated	50.6 (45.3–55.9)	55.4 (51.3–59.4)	50.1 (46.9–53.3)	49.0 (44.9–53.2)	48.6 (44.7–52.5)	.02
Intermediate	200–239 or treated to goal	39.3 (34.5–44.4)	34.5 (30.9–38.1)	38.5 (35.1–42.1)	36.2 (33.0–39.5)	36.7 (32.3–41.3)	
Poor	≥240	10.1 (8.3–12.4)	10.2 (8.2–12.5)	11.4 (9.5–13.5)	14.8 (12.7–17.1)	14.7 (11.5–18.6)	
**Hemoglobin A_1c_, %**							
Mean	NA	5.7 (5.6–5.7)	5.6 (5.6–5.7)	5.5 (5.5–5.6)	5.6 (5.5–5.6)	5.6 (5.5–5.7)	.01
Ideal	<5.7 untreated	66.1 (61.1–70.9)	70.8 (67.8–73.7)	74.3 (71.6–76.8)	72.9 (70.1–75.5)	65.8 (59.7–71.4)	.008
Intermediate	5.7–6.4 or treated to goal	30.2 (25.5–35.3)	26.0 (23.1–29.1)	23.2 (21.0–25.6)	24.5 (22.2–26.9)	30.8 (25.6–36.6)	
Poor	≥6.5	3.7 (2.5–5.4)	3.2 (2.4–4.4)	2.5 (1.7–3.7)	2.6 (1.9–3.5)	3.4 (2.3–4.9)	

Abbreviations: CI, confidence interval; CVH, cardiovascular health; DBP, diastolic blood pressure; NA, not applicable; SBP, systolic blood pressure.

a Component definitions and scoring used were those previously described by Lloyd-Jones et al. with modification of hemoglobin A_1c_ as a proxy for fasting plasma glucose (13). The specific definitions used in this analysis are presented.

b *P* value calculated from adjusted Wald or Pearson’s χ^2^ tests that were corrected for the survey design.

c The CVH score comprises 7 components: smoking, body mass index, diet, physical activity, blood pressure, total cholesterol, and hemoglobin _A1c_ (used as a proxy for fasting plasma glucose) (13). Each component was scored as ideal (2 points), intermediate (1 point), or poor (0 points) based on guidelines described by Lloyd-Jones et al (13). The continuous overall CVH score was calculated by summing the 7 components scores. Ideal CVH was defined as meeting ideal criteria for 5 to 7 of the components.

d American Heart Association Healthy Diet Score includes ≥4.5 cups of fruits or vegetables a day; two 3.5-ounce servings of fish per week; ≥3 one-ounce equivalent servings of whole grains per day; <1,500 mg of sodium per day; ≤36 ounces of sugar-sweetened beverages per week.

In the multivariable logistic regression models, we found a consistent association between very short sleep duration and decreased odds of ideal CVH (Table 3). In the unadjusted model, those with very short sleep duration had 0.53 times the odds of ideal CVH (95% CI, 0.39–0.72) as those who slept 7 to <8 hours. We also saw an association between very long sleep duration and decreased odds of ideal CVH (0.75; 95% CI, 0.58–0.98). We found no consistent association with ideal CVH for the other sleep durations. After adjusting the models for demographic factors and then social and clinical factors, similar associations were found. In the fully adjusted model, the effect was slightly attenuated but still significant with an adjusted odds ratio (OR) of 0.65 (95% CI, 0.47–0.90). The association between very long sleep duration and ideal CVH remained similar with an adjusted OR of 0.72 (95% CI, 0.55–0.94).

**Table 3:** Association Between Sleep Duration Categories and Ideal CVH in Sequential Adjusted Logistic and Linear Regression Models Among US Adults Aged 20–75 (N = 7,784), National Health and Nutrition Examination Survey, 2013–2016

Sleep Duration, No. of Hours	Model 1^a ^Estimate (95% CI)	Model 2^b ^Estimate (95% CI)	Model 3^c ^Estimate (95% CI)	Model 4^d ^Estimate (95% CI)
**Odds of ideal CVH**				
<6	0.53 (0.39 to 0.72)	0.63 (0.45 to 0.87)	0.56 (0.41 to 0.77)	0.65 (0.47 to 0.90)
6 to <7	0.90 (0.76 to 1.07)	0.97 (0.80 to 1.18)	0.91 (0.76 to 1.09)	0.97 (0.80 to 1.19)
7 to <8	1 [Reference]			
8 to < 9	1.03 (0.82 to 1.28)	0.95 (0.74 to 1.23)	1.04 (0.84 to 1.30)	0.96 (0.75 to 1.23)
≥9	0.75 (0.58 to 0.98)	0.70 (0.53 to 0.93)	0.78 (0.60 to 1.02)	0.72 (0.55 to 0.94)
**Mean differences in CVH score, mean**				
<6	−0.80 (−1.04 to −0.55)	−0.48 (−0.69 to −0.27)	−0.69 (−0.94 to −0.45)	−0.41 (−0.61 to −0.20)
6 to <7	−0.31 (−0.45 to −0.17)	−0.21 (−0.34 to −0.08)	−0.3 (−0.44 to −0.16)	−0.2 (−0.33 to −0.06)
7 to <8	[Reference]			
8 to < 9	−0.15 (−0.36 to 0.06)	−0.18 (−0.39 to 0.03)	−0.12 (−0.32 to 0.07)	−0.16 (−0.36 to 0.03)
≥9	−0.51 (−0.78 to −0.24)	−0.38 (−0.63 to −0.13)	−0.45 (−0.70 to −0.19)	−0.33 (−0.57 to −0.09)

Abbreviations: CI, confidence interval; CVH, cardiovascular health; OR, odds ratio.

a Model 1: Unadjusted.

b Model 2: Adjusted for demographic factors of weighted age quartiles, sex, race/ethnicity, education level, and family income-to-poverty ratio category.

c Model 3: Adjusted for social and clinical factors of depression status, binge alcohol use, and prescription sleep aid use.

d Model 4: Fully adjusted model including factors from Models 2 and 3.

When examining the CVH score as a continuous variable, findings in the unadjusted and adjusted models were similar. In the fully adjusted model, a significant decrease was observed in the mean CVH score for participants with very short (−0.41; 95% CI, −0.61 to −0.20) and very long (−0.33; 95% CI, −0.57 to −0.09) sleep durations, compared with those with sleep duration of 7 to <9 hours. Additionally, short sleep duration (6 to <7 hours) was also associated with a significant decrease in mean CVH score; no significant association was found for those with sleep duration of 8 to <9 hours.

## Discussion

Ours is the first description to our knowledge of an association between sleep duration and ideal CVH that used a nationally representative sample of US adults. In this cross-sectional evaluation, very short sleep of <6 hours and very long sleep of ≥9 hours were associated with decreased odds of ideal CVH and a significant decrease in mean CVH score after adjusting for demographic, clinical, and social factors. Because ideal CVH is related to the future risk of CVD, these findings provide evidence of associations between sleep duration and ideal CVH that require further investigation, including exploring causal associations that we were unable to assess in this cross-sectional study.

The overall prevalence of ideal CVH in this population is similar to past descriptions of adults. Globally, the prevalence of ideal CVH status overall is approximately 20% with estimates of between 15% and 20% for North American adults (23), which is similar to what we observed in our study. The proportion of US adults in our study sleeping 7 to <9 hours per night is consistent with prior estimates. Data from the Behavioral Risk Factor Surveillance System and the National Health Interview Survey estimated that about 60% of adults reported a sleep duration of this length (2,24), consistent with the estimated 59.6% of adults in our study.

Data on the association between sleep duration and ideal CVH is scarce, though some work has been done looking at sleep debt, sleep quality, and daytime sleepiness. In a sample of older women (mean age 72 years), sleep debt was associated with poor CVH, even after accounting for potentially confounding demographic and socioeconomic factors (16). Poor sleep quality in a population of Ecuadoran adults, as measured by the Pittsburgh Sleep Quality Index (25), was associated with some components of the CVH metrics but was not associated with poor CVH status overall (26). In that same population, excessive daytime sleepiness was also not associated with poor CVH status (27). This is in contrast to substantial evidence of an association between incident cardiovascular disease and short sleep duration and, to a lesser extent, long sleep duration (5,6).

The relationship between sleep and CVH likely differs from the association between sleep and CVD previously described. The etiology behind the differences is unclear but may be related to a bi-directional association between sleep patterns and a person’s CVH metrics. The biologic mechanism for the effect of sleep duration on CVH has multiple pathways. Sleep duration is independently associated with several CVH metrics, such as weight status and hypertension, and with insulin sensitivity, which can lead to type 2 diabetes (5,6,28). Poor sleep may also lead to changes in behavioral components such as increased frequency of smoking or decreased physical activity (29). It is still unclear, however, whether less than ideal health in these components may also affect sleep duration in the reverse manner. Also unclear is whether ideal CVH can lead to better sleep quality.

We found evidence of associations between both very short and very long sleep duration and decreased odds of ideal CVH and a significant decrease in mean CVH score. Although the U-shaped association between sleep duration and CVD has been increasingly described (4–6,30), some studies have shown that long sleep duration is protective for CVD or found nonsignificant associations (30). The biologic mechanism of long sleep duration and CVD or CVH is not as well understood as the mechanism of short sleep duration, especially because observed associations with long sleep durations may be related to reverse causality (eg, a result of CVD) or subclinical disease (30). We did adjust for some possible explanatory factors such as depression and use of prescription sleep aids, but the potential for residual confounding or other bias may have affected the results in this study.

Our study had several strengths, including the large sample size and use of the nationally representative NHANES population. However, our study had limitations. Although the sample size was large enough to detect significant differences, we excluded a proportion of participants (1,468 of 9,252 [16%] eligible adults without prevalent CVD) because of missing data who otherwise would have been eligible for inclusion. However, when comparing all participants with available data to the analytic sample, we found no substantial differences. Sleep duration was assessed by self-report and included only assessment of weekday or workday sleep. In past studies, participant self-reported sleep durations were overestimated compared with objectively measured durations, especially by those with the shortest sleep durations (31). Thus, misclassification is possible, though the direction of bias is likely toward the null because participants tended to overestimate instead of underestimate sleep. Additionally, several of the CVH metrics were also self-reported, including smoking status, physical activity, and dietary habits. The other components of the CVH metrics, however, were measured in a standardized manner with trained assessors as part of the NHANES data collection process. The cross-sectional study design does not allow for inferring causality between sleep duration and ideal CVH status. Finally, we could not account for other confounders such as sleep apnea and use of nonprescription sleep aids in our analysis.

Our study demonstrated an association between very short and very long sleep duration and reduced CVH, as suggested by decreased odds of ideal CVH and a decrease in mean CVH score. More work is needed to understand the implications of sleep duration and the metrics of CVH, including potential causal associations.

## Post-Test Information

To obtain credit, you should first read the journal article. After reading the article, you should be able to answer the following, related, multiple-choice questions. To complete the questions (with a minimum 75% passing score) and earn continuing medical education (CME) credit, please go to http://www.medscape.org/journal/pcd. Credit cannot be obtained for tests completed on paper, although you may use the worksheet below to keep a record of your answers.

You must be a registered user on http://www.medscape.org. If you are not registered on http://www.medscape.org, please click on the “Register” link on the right hand side of the website.

Only one answer is correct for each question. Once you successfully answer all post-test questions, you will be able to view and/or print your certificate. For questions regarding this activity, contact the accredited provider, CME@medscape.net. For technical assistance, contact CME@medscape.net. American Medical Association’s Physician’s Recognition Award (AMA PRA) credits are accepted in the US as evidence of participation in CME activities. For further information on this award, please go to https://www.ama-assn.org. The AMA has determined that physicians not licensed in the US who participate in this CME activity are eligible for *AMA PRA Category 1 Credits™*. Through agreements that the AMA has made with agencies in some countries, AMA PRA credit may be acceptable as evidence of participation in CME activities. If you are not licensed in the US, please complete the questions online, print the AMA PRA CME credit certificate, and present it to your national medical association for review.

## Post-Test Questions

### Study Title: Association Between Sleep Duration and Ideal Cardiovascular Health Among US Adults, National Health and Nutrition Examination Survey, 2013–2016

#### CME Questions

1. You are seeing a 49-year-old man who complains of fatigue. He works 2 jobs and is getting about 5 hours of sleep per night. In general, what can you tell him about sleep duration and the risk for cardiovascular disease (CVD)?
  - Incident CVD is associated with average sleep duration less than 6 hours per night only
  - Incident CVD is associated with average sleep duration less than 7.5 hours per night only
  - Incident CVD is associated with both abnormally short and abnormally long sleep durations
  - The incidence of CVD does not appear related to sleep duration
2. The patient feels guilty about not getting enough sleep. What was the proportion of adults who slept between 7 and less than 8 hours per night in the current study?
  - 15%
  - 30%
  - 50%
  - 65%
3. The patient has a number of negative cardiovascular risk factors. The recommended number of hours of sleep was most closely associated with which one of the following variables of ideal CVH?
  - Physical activity for at least 150 minutes per week
  - Body mass index less than 25 kg/m^2^
  - Healthy diet
  - Blood pressure lower than 120/80 mm Hg
4. Compared with sleep duration between 7 and less than 8 hours per night, which one of the following average sleep durations was significantly associated with decreased odds of ideal CVH in the current study?
  - None: There was no difference in the rate of ideal CVH based on sleep duration
  - Only sleep duration less than 6 hours was associated with a lower rate of ideal CVH
  - Only sleep duration of at least 9 hours was associated with a lower rate of ideal CVH
  - Very short or very long sleep durations were associated with a lower rate of ideal CVH
